# Contribution of the Tyr-1 in Plantaricin149a to Disrupt Phospholipid Model Membranes

**DOI:** 10.3390/ijms140612313

**Published:** 2013-06-07

**Authors:** José L. S. Lopes, Maria J. Gómara, Isabel Haro, Georgina Tonarelli, Leila M. Beltramini

**Affiliations:** 1 Institute of Physics of Sao Carlos, University of Sao Paulo, Av. Trabalhador Saocarlense 400, Sao Carlos, SP 13560-970, Brazil; E-Mail: zeluiz@ifsc.usp.br; 2 Institute of Advanced Chemistry of Catalonia (IQAC-CSIC), Barcelona 08034, Spain; E-Mails: mariajose.gomara@iqac.csic.es (M.J.G.); isabel.haro@iqac.csic.es (I.H.); 3 Department of Organic Chemistry, National University of the Litoral, Santa Fe C.C.242 (3000), Argentina; E-Mail: tonareli@fbcb.unl.edu.ar

**Keywords:** antimicrobial peptide, membrane models, peptide-lipid interaction, plantaricin

## Abstract

Plantaricin149a (Pln149a) is a cationic antimicrobial peptide, which was suggested to cause membrane destabilization via the carpet mechanism. The mode of action proposed to this antimicrobial peptide describes the induction of an amphipathic α-helix from Ala7 to Lys20, while the *N*-terminus residues remain in a coil conformation after binding. To better investigate this assumption, the purpose of this study was to determine the contributions of the Tyr1 in Pln149a in the binding to model membranes to promote its destabilization. The Tyr to Ser substitution increased the dissociation constant (*K*_D_) of the antimicrobial peptide from the liposomes (approximately three-fold higher), and decreased the enthalpy of binding to anionic vesicles from −17.2 kcal/mol to −10.2 kcal/mol. The peptide adsorption/incorporation into the negatively charged lipid vesicles was less effective with the Tyr1 substitution and peptide Pln149a perturbed the liposome integrity more than the analog, Pln149S. Taken together, the peptide-lipid interactions that govern the Pln149a antimicrobial activity are found not only in the amphipathic helix, but also in the *N*-terminus residues, which take part in enthalpic contributions due to the allocation at a lipid-aqueous interface.

## 1. Introduction

Several antimicrobial peptides (AMPs) are able to simultaneously attack various microorganisms (including bacteria, fungi, and enveloped viruses [1,2]) and exhibit cytotoxic activity towards tumor cells [3]. The search for new compounds to be used in the pharmaceutical/food industries for controlling bacterial growth and providing food safety [4,5] has increased interest in this group of natural peptides that can inhibit microorganism growth with a bactericidal or bacteriostatic mode [6–8]. AMPs were reported as natural antibiotics [9], modulators of immune activities [10], promising candidates as natural food preservatives [11], and are currently being investigated in therapeutic use [12] for controlling sepsis that causes high newborn morbidity, and neonatal mortality, in developed and developing countries [13,14].

Although AMPs have been identified from different sources, the bacteriocins [15] are one of the most investigated groups, mainly the members isolated from lactic acid bacteria (LAB) [16–18], which were shown to vary widely in size, primary and secondary structures [19,20], but at the same time share some common features: cationicity, amphiphilicity, and act by altering the permeability of cellular membranes [21,22]. From the LABs, a widely studied AMP is Plantaricin A (PlnA) [23–26], a 26-residue peptide (KSSAYSLQMGATAIKQVKKLFKKWGW) produced by *Lactobacillus plantarum* C11, which displays antimicrobial activity against certain sensitive strains by membrane permeabilization, although its primary function is considered to act as a peptide pheromone, controlling the production of antimicrobial peptides in *Lactobacillus plantarum* C11. Both are differential activities with different bacterial strains. In the binding to lipid membranes, PlnA adopts a helical structure that is essential for both the pheromone function and antimicrobial activity. The antimicrobial activity of PlnA was shown to be unspecific [26] and with high MIC values reported, since the natural function of the peptide was suggested to act as a pheromone. In a similar way to PlnA, the peptide Plantaricin149 (Pln149), produced by *L. plantarum* NRIC 149, is also cationic in nature, composed of 22 amino acid residues with inhibitory activity against some pathogenic bacteria [27,28] with high minimum inhibitory concentration (MIC) values [27,29], compared to the MIC of other LAB bacteriocins [26]. The mode of action proposed to the amidated analog, Pln149a, (charge +7 at pH 7.0) assumes it adopts an unordered conformation in aqueous solution [28,29]; however, upon binding to negatively charged membranes, Pln149a changes to an α-helix conformation. The molecular model of the whole Pln149a sequence, created by SP^3^ software, is a helix structure which was proposed to extend from the residue Ala7 to Lys20 [28] in an amphipathic structure with the polar residues K11, K14, K15, K18, and K19 of Pln149a along one side of the helix and the nonpolar residues, I10, V13, L16, and F17 along the other. A helical conformation was observed in the peptide’s structure when in the presence of sodium-bis-(2-ethylhexyl)-sulfosuccinate (AOT) micelles, trofluoroethanol (TFE) [29], and negatively charged vesicles [30]. This helix was proposed to be incorporated onto the membrane surface via electrostatic interactions, covering the outer surface of the membrane in a “carpet”-like manner [30]. The kinetics adsorption of Pln149a on DPPG monolayers showed that the lipid packing was increased by the presence of the peptide and the monolayer disruption occurred with a behavior dependent of the peptide concentration. After a critical concentration of peptide (~2.5 μM) was reached, the mixed system (peptide and lipids) yield an elevated dilatational elasticity (E) modulus and was followed by a steep decrease in the E values, which indicated an increase in the fluidity of the model membrane than can be attributed to the disruption of the monolayer by a solubilization mechanism [29].

The *N*-terminus residues of Pln149a remained in coil conformation and do not belong to the amphipathic helix, but the removal of this portion was shown to affect the mechanism of action and the bactericidal properties of the peptide [31], suggesting the incorporation of the *N*-terminus residues within the external phospholipid bilayer to promote the total membrane disruption. In this paper, peptides analogs to Pln149 were synthesized to investigate the contributions of the Tyr residue at position 1 in the interaction of the peptide with model membranes, in order to validate the proposed mechanism for Pln149a [29] and to determine the nature of the interactions of the *N*-terminus residues of Pln149a with phospholipids, since an important enthalpic contribution was observed with the presence of the Tyr1 at this position on the antimicrobial peptide. In addition to the Y1S substitution, two different Pln149-analogs were synthesized with the incorporation of a Trp residue at position 17 (replacing the original Phe17) in order to better observe small differences in the binding of the antimicrobial peptide in membranes due to the single *N*-terminus substitution in Pln149a.

## 2. Results and Discussion

To better understand the mode of action of an AMP, this work has investigated the interactions of the peptide Pln149a with phospholipid vesicles. The binding of Pln149-analogs containing the Tyr1 was promoted onto vesicles and compared with the binding of analogs in which a Tyr-to-Ser substitution was performed.

### 2.1. Peptide Synthesis, Purification, and Characterization

Four Pln149 analogs were synthesized by an Fmoc strategy to investigate the binding of this antimicrobial peptide to model membranes. The first analog was Pln149a, the *C*-terminus amidated version of the natural Pln149 antimicrobial peptide, as previously described [28,29]. The second analog was Pln149S, a peptide in which the Tyr residue at position 1 (*N*-terminal end) was substituted by a Ser residue (Y1S substitution). In the two other analogs synthesized, the introduction of a Trp residue was performed at position 17 (replacing the original Phe17). These analogs were named Pln149W and Pln149SW, in which a F17W and both Y1S-F17W substitutions were introduced, respectively. Edman degradation and MS analyses determined the correct primary structure and molecular masses of 2424.0 Da for Pln149a, 2347.9 Da for Pln149S, 2479.5 Da for Pln149W, and 2403.6 Da for Pln149SW. Peptides were subsequently purified by reverse-phase chromatography (RPC), with major peaks eluted at of 38%, 36%, 46%, and 42% on the ACN gradient, respectively (Supplementary Figure S1). After RPC, the degree of purity of the peptides was >95%.

### 2.2. 8-Aminonaphthalene-1,3,6-Trisulfonic Acid (ANTS)-P-Xylene-Bis-Pyridinium Bromide (DPX) Leakage Assays

**Figure 1:**
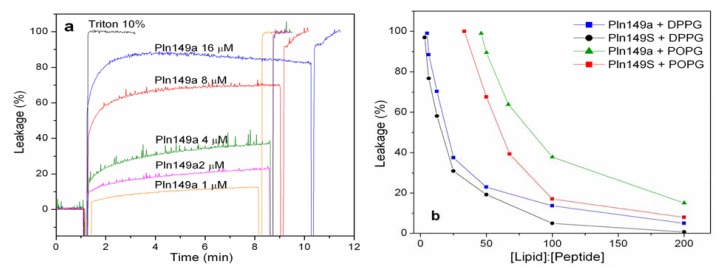
Leakage assays with Plantaricin149a (Pln149a) and Pln149S. (**a**) The internal content of DPPG liposomes was released by the action of Pln149a (1 to 16 μM). Total leakage (100%) was achieved with the addition of 10% Triton-X; (**b**) Comparative leakage curves of Pln149a and Pln149S with DPPG and POPG liposomes.

Leakage experiments were performed to assess the membrane perturbation induced by the presence of Pln149a and Pln149S. As shown in Figure 1a, the leakage of the DPPG liposomes encapsulating ANTS-DPX increased with the increment of the Pln149a concentration in solution. Initially, the addition of small amounts (up to 2 μM) of Pln149a promoted an increase in the values of leakage in a nearly linear dependence. The value of 23% leakage was reached when the lipid to peptide 50:1 molar ratio was used. Reducing the lipid to peptide molar ratio from this value on, promoted a significant increase in the leakage observed in the liposomes, but still, elevated concentrations of Pln149a (16 μM) were necessary to cause 100% leakage at the early time of incubation with the liposomes. The results of the leakage promoted by Pln149a to the DPPG vesicles are in agreement with the disruption of the phospholipid monolayers [29], which occurred at concentrations of 2.5 μM to a pure DPPG monolayer.

The leakage caused by the analog with the Tyr to Ser substitution (Pln149S) was less than 4% on the same DPPG vesicles at lipid to peptide molar ratios higher than 100:1 (Supplementary Figure S2A). The leakage action for the Tyr-substituted analog effectively started at 50:1 lipid to peptide ratio (causing a 19% leakage), and then followed the same concentration-dependent behavior described for Pln149a.

In an attempt to compare the leakage action of both peptides in a more fluid membrane model, the same assay was conducted with POPG vesicles. The lipid to peptide molar ratio required to promote an effective leakage on POPG liposome was significantly higher than for DPPG liposomes for both Pln149a and Pln149S (Supplementary Figure S2B,C). With this more fluid vesicle, the leakage action of Pln149a was 16% at a lipid to peptide ratio of 200:1, and reached 100% leakage at around a 66:1 ratio. The values obtained for the leakage of Pln149a in the POPG vesicles is well correlated with the values of other AMPs, like Magainin (M2a), a peptide isolated from amphibians, that promotes a leakage of 50% in DOPC, DOPG and mixed vesicles of these two phospholipids at a 90:1 lipid to peptide ratio [32].

Comparing the leakage curves of Pln149a and Pln149S after 500-s interaction with the liposomes of DPPG and POPG (Figure 1b), it is possible to observe the effect of Y1S substitution to promote the reduction of the lytic properties of the peptide, where approximately twice the Pln149S were required to cause a leakage similar to that promoted by Pln149a. For Pln149S, 18% leakage was reached at a 100:1 lipid to peptide ratio and the 100% leakage occurred at a 33:1 ratio.

Additionally, the assays to check the hemolytic activity of both Pln149a and Pln149S showed no lytic effect (less than 5%, Supplementary Figure S2) in the concentration range studied here (1 to 500 μM), suggesting their low toxicity to human erythrocytes, which presents the major composition of zwitterionic phospholipids at the cell membrane.

Taken together, these results strongly support the idea that both Pln149a and Pln149S may be associated with the polar head group of the phospholipids with an initial electrostatic interaction driven by the region of the amphipathic helix induced on the peptide, in a very similar way described to PlnA [24–26]. However, the efficiency of this incorporation can also depend on the presence of hydrophobic residues at the *N*-terminus of the peptide (Tyr1), since this substitution affects the hydrophobic moment of the peptide, which is also an important feature that contributes to the lytic effect.

### 2.3. Far-UV Circular Dichroism Analyses

**Figure 2:**
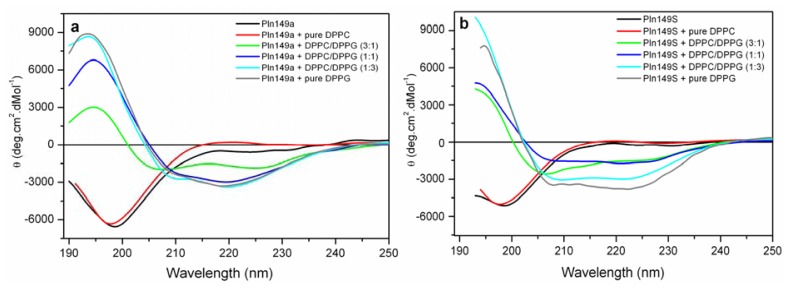
Far-UV CD spectra of Pln149a and Pln149S in the presence of mixed vesicles of DPPC/DPPG. Measurements were taken from 190 to 250 nm as the average of 8 scans at 25 °C using a 0.1-cm-path-length quartz cuvette with (**a**) Pln149a and (**b**) Pln149S (0.15 mg/mL), both peptides in water (black), incubated with pure DPPC (red), DPPC/DPPG (3:1) (blue), DPPC/DPPG (1:1) (magenta), DPPC/DPPG (1:3) (green), and pure DPPG (grey) liposomes.

The CD spectra of Pln149a and Pln149S confirmed these peptides are completely disordered in aqueous solution, with a strong negative peak between 195 and 198 nm [33]. This is similar to PlnA, which does not present helical structure in aqueous solution. However, when incubated with different compositions of DPPC/DPPG vesicles, an α-helix induction was observed on the secondary structure of both peptides (Figure 2), which spectra present the two negative minima at 208 and 222 nm and a positive peak at 196 nm. Similar behavior was also observed in PlnA [23–26] and other linear cationic peptides when binding to negatively charged membranes [30], evidencing the importance of electrostatic interactions to drive the initial binding of the AMP to the target.

Disorder-to-order transitions in the CD spectra of both Pln149a and Pln149S were observed with the increase of the density of the negative charge on the liposome, which could reflect the increasing amount of peptide bound to surface of the vesicles. Additionally, at the CD spectra with the lowest content of DPPG (25% and 50% in the mixed vesicles), it was possible to observe that the intensity at 222 nm for Pln149a are higher than observed for Pln149S. Therefore, it can be inferred that Y1S substitution also affected the amount of peptide bound in the membranes, especially when a low content of negative phospholipids is present.

### 2.4. Liposome Titration into Pln149-Analogs with a Trp Residue

Two different analogs were synthesized with a Trp residue incorporated into the primary sequence of Pln149 to investigate the binding of these peptides to phospholipid vesicles, using assays of intrinsic fluorescence emission to monitor the relative increase of the fluorescence of the quantum yield and the λ_max_ of the Trp residue.

**Figure 3:**
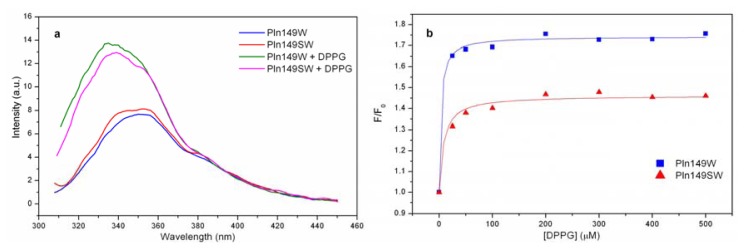
Tryptophan fluorescence titration (**a**) Fluorescence emission spectra of Pln149W and Pln149SW in aqueous solution and in the presence of DPPG vesicles (at a 1:500 peptide to lipid ratio); (**b**) Data fit at 350 nm of the Tryptophan titration assay of Pln149W and Pln149SW to a hyperbolic equation.

Figure 3a shows the fluorescence emission spectra for both Pln149W and Pln149SW in aqueous solution, in which the Trp residue is exposed to interaction with aqueous solution because the λ_max_ are centered at 350 nm, as expected for this residue free in solution (350–355 nm) [34]. However, a 10 nm blue shift was observed in the λ_max_ of both peptides when in contact with the LUVs of DPPG due to the local rearrangement of the Trp residue, which was allocated in an environment less exposed to the aqueous solution. This blue shift was noted in all lipid/peptide ratios tested.

The data at 350 nm from the titration of the LUVs into the peptide solution were fitted with a hyperbolic equation, which provided the values for *K*_D_ of 57.1 nM and 146 nM for Pln149W and Pln149SW, respectively, showing the high affinity of these molecules for negative surfaces like the pure DPPG liposomes. Additionally, it was possible to observe that Pln149W binds approximately three times more strongly than Pln149SW, reflecting once again the involvement of the Tyr residue at the *N*-terminal end in the binding to the LUVs. The fraction of bound peptide on the surface of the DPPG liposomes also reflects the high affinity of Pln149W and Pln149SW to the negative phospholipid. Nearly 90% of the Pln149W was bound to the LUVs at the first concentration of phospholipid used (1:25 peptide/lipid molar ratio). During the titration, the amount of peptide bound to the liposomes increased proportionally to the increase of the concentration of lipid in the solution, reaching almost 100% binding. The values of the fraction of bound peptides during the entire titration are in Table 1.

The comparative study of the two Trp-analogs of Pln149a also showed the involvement of the Tyr1 in the binding of the antimicrobial peptide. Nonetheless, the conclusions based on the Trp-analogs apply only to the Trp mutants, owning to the fact the binding mode of the Trp analogs could be different from that of the native Pln149a.

### 2.5. Isothermal Titration Calorimetry (ITC) Experiments

**Figure 4:**
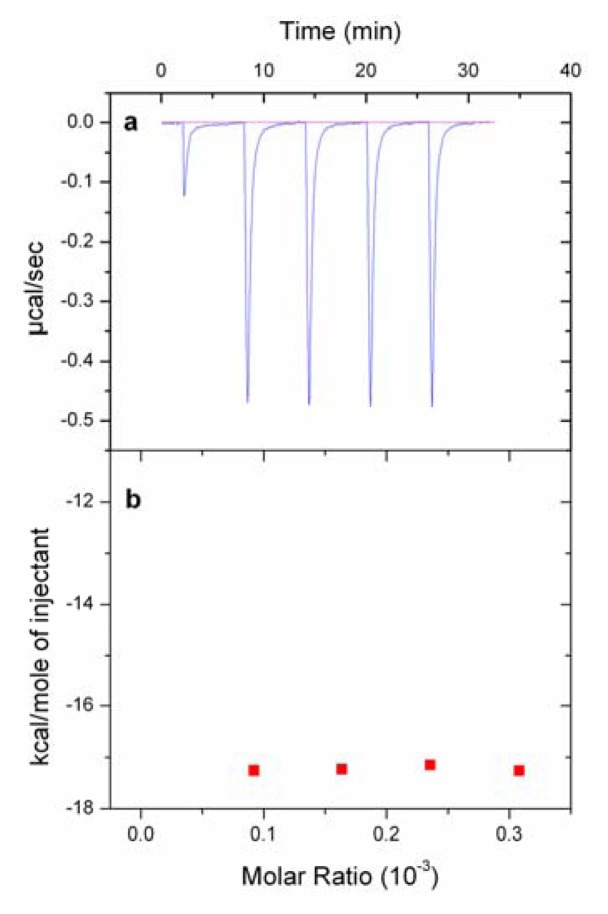
Determination of the enthalpy of binding of Pln149a to the DPPG vesicles. (**a**) Titration of Pln149a aliquots (100 μM) in the calorimeter cell containing DPPG (10 mM) vesicles at 25 °C; (**b**) Heat of reaction of each injection, determined by the integration of the peaks showed in (**a**).

To check whether the difference in the binding of the analogs with/without the Tyr1 was reflected in their thermodynamic parameters, the total enthalpy of binding of the peptides when interacting with DPPG vesicles was calculated under the assumption that all injected peptide was bound to the lipid vesicle surface. As shown in Figure 4a, the binding of Pln149a to LUVs of DPPG was an exothermic reaction in which each injection promoted the liberation of the heat reaction, with a peak intensity of approximately −0.475 μcal/s in the titrations performed. Integrating the peaks (Figure 4B), the binding of Pln149a to the vesicles gave an enthalpy of binding (Δ*H*) of −17.2 kcal/mol. The value determined for the enthalpy of binding of Pln149a to DPPG vesicles was similar to the values described of several AMPs binding to membrane models. For instance, the interaction of M2a, from the Magainin class, with POPC vesicles is followed by a Δ*H* of −18.4 kcal/mol [35], and also the titration of the peptide βAP(1–40) with POPC/POPG (75/25 mol/mol) vesicles gives a Δ*H* of −14 kcal/mol [36].

The Y1S substitution was reflected in the reduction of the peak intensity (−0.300 μcal/s) that, after integrations, gave a Δ*H* of −10.6 kcal/mol to the binding of the Pln149S peptide to the DPPG vesicles. Because Pln149a and Pln149S share identical primary structures (except for the amino acid residue at position 1) and Δ*H*_Pln149a_ was approximately 40% higher than Δ*H*_Pln149S_, it is possible to confirm that the Tyr1 residue is responsible for an important role in the enthalpic contributions during the peptide binding to liposomes. Moreover, the decrease observed with the Y1S substitution probably reflects the weakening of the interactions between the peptide at the lipid-aqueous interface, where the Tyr residue is more likely to be located. The stronger binding properties observed to Pln149a than to Pln149S could be associated to the fact that the Tyr residue within the polypeptide chain associates strongly with phospholipids in membrane models [37,38]. In membrane proteins, for instance, this residue is not randomly distributed in the protein structure, but instead, it has a preferential distribution, being located mainly in regions that board the transmembrane segments of the protein [39,40], especially at the lipid-aqueous interface.

The ITC experiment was performed here in a condition that allows assuming that all the injected peptide was bound to the lipid vesicles, and then enthalpy of binding could be determined. A more complete isotherm of the binding of Pln149a to different liposomes might provide more details to the mechanism of action of this peptide.

### 2.6. Antimicrobial and Hemolytic Activities

The MIC and MBC values of the peptides against the two bacteria are in Table 2. Although Pln149a and Pln149S presented the same MIC value against *S. aureus* (19 μM), a difference in action was observed. In the wells below the MIC, Pln149a presented a gradual decrease of inhibition (78%, 61%, 43%), while Pln149S presented 98% inhibition in the presence of 19 μM (44 μg/mL), however, only 9% inhibition was observed in the following dilution (38 μM). This difference was also observed in the MBC values of 78 and 155 μM for Pln149a and Pln149S, respectively, which shows Pln149a as the most active antimicrobial peptide due to the higher bactericidal effect. Similar behavior was observed to the peptide’s antimicrobial activity against *P. aeruginosa*, which presented the same MIC value of 310 μM for Pln149a and Pln149S (369 and 363 μg/mL, respectively), and a more elevated MBC to Pln149S (310 μM) than to Pln149a (155 μM). Similar to PlnA, Pln149a could be considered a weak antimicrobial agent due to the high MIC values found here, and as already described of this peptide [28,29,31]. Nevertheless, the motivations for studying the mechanism of action of this antimicrobial peptide against different classes of microorganisms are: its stability in acid pH ranges, its inactivation by humans digestive proteases (which is of particular interest for suggesting its application as a nontoxic and safe food preservative to control the microflora of fermented foods, with no effect against the microflora of the human intestine). Also, the study of Pln149a presents an alternative to correlate/validate biophysical studies with model membranes with its antimicrobial activity, with the design of a peptide with inhibitory activity potentiated, by adding modifications at the peptide ends of the peptide and/or designing chimerical peptides with the most important parts of Pln149 and other AMPs.

It has been already reported that the binding of Pln149a to lipids can be correlated to its antimicrobial activity. The binding of Pln149a to model membranes constructed with different zwitterionic and negatively charged phospholipids [29] lipids showed that in the presence of a negative density of charge at the membrane surface a strong interaction is observed between the peptide and the lipid. In agreement with that, the peptide is more active against Gram-positive bacteria, which presents major membrane composition of negatively charged phospholipids, than against microorganisms with major membrane composition of zwitterionic phospholipids [27–29,31]. Additionally, in the present manuscript, the peptide Pln149a presented higher bactericidal effect than Pln149S against the two microorganisms employed (*S. aureus* and *P. aeruginosa*), in good correlation with the strongest binding of the peptide Pln149a to the lipids observed in the ITC results and leakage assays, where Pln149a was the peptide with the highest enthalpy of binding and the highest leakage action, respectively.

In the hemolytic assay, both Pln149a and Pln149S presented low hemolytic activity (below 5%) from 1 to 500 μM. Although it was possible to note that the Y1S substitution slightly reduced the hemolytic effect of the antimicrobial peptide, suggesting that Tyr residue in the peptide Pln149a may be involved in its incorporation to the lipid bilayers. The involvement of the Y166 in lipid binding was demonstrated to the synthetic peptide from apoA-I when associating to lipids [41].

## 3. Experimental Section

### 3.1. Peptide Synthesis and Purification

Four Pln149-derived peptides were manually synthesized by solid-phase with the Fmoc strategy on Rink Amide Tentagel R Ram. The names and primary structures of the synthesized Pln149-peptides are Pln149a (YSLQMGATAIKQVKKLFKKKGG), Pln149S (SSLQMGATAIKQVKKLFKKKGG), Pln149W (YSLQMGATAIKQVKKLWKKKGG), and Pln149SW (SSLQMGATAIKQVKKLWKKKGG). The Fmoc groups on the resin and on the amino acid *N*-terminus were removed by treatment with 20% piperidine in dimethylformamide (DMF) until a negative Kaiser assay [42] result was obtained. Coupling reactions were promoted with Fmoc-protected amino acids (3 equiv.), HATU (2-(1H-7-azabenzotriazol-1-yl)-1,1,3,3-tetramethyl uranium hexafluorophosphate methanaminium, 3 equiv.) and DIEA (*N*-Etildiisopropilamine, 3 equiv.) in DMF (1/1, volume ratio), and the efficiency >95% was checked by Kaiser test. After the incorporation of all amino acid residues, the peptides were cleaved with a mixture of trifluoroacetic acid (TFA):triisopropylsilane(TIS):ethanedithiol(EDT):H_2_O (94:1:2.5:2.5, *v*/*v*) for 4 h. The crude peptides were precipitated with cold diethyl ether, centrifuged at 3000 rpm for 5 min and lyophilized. Purification of the crude peptides was performed by reverse-phase chromatography using an ÄKTA Purifier System (GE Healthcare, Upsala, Sweeden) with an YMC-Pack Polymer C_18_ column (250 × 4.6 mm, 6 μm bead size, Waters, Milford, MA, USA) equilibrated with 0.1% (*v*/*v*) TFA/water (solvent A). Elution was performed at a 1 mL/min flow rate using a two-step gradient: initially, from 0% to 70% solvent B (acetonitrile 90% in water containing 0.1% TFA) over 40 min, followed by 70%–100% of the same solvent over 1 min. Absorbance was monitored at 220 and 280 nm. The purified peptides were analyzed by mass spectrometry (MS) and automated Edman degradation. Peptide concentration was determined by UV absorbance at λ = 280 nm for Pln149a, Pln149W and Pln149SW using an extinction coefficient (ɛ_280_) of 1490, 6990, 5500 M^−1^ cm^−1^, respectively. The values obtained were very similar to the ones determined by Lowry method for the peptides Pln149a, Pln149S, Pln149W and Pln149SW.

### 3.2. Liposome Preparation

Large unilamellar vesicles (LUVs) of dipalmitoyl-phosphatidyl-glycerol (DPPG) and palmitoyl-oleoyl-phosphatidyl-glycerol (POPG) were prepared by the extrusion method, as described elsewhere [29]. LUVs of these two phospholipids containing encapsulated probes were prepared as follows. The required amounts of dry phospholipids were dissolved in a mixture of chloroform/methanol (2:1), and solvent was slowly removed by evaporation in the presence of nitrogen to form a thin lipid film, followed by lyophilization for 1 h. Lipid films were resuspended in 5 mM Hepes (pH 7.4) containing 20 mM NaCl, 12.5 mM 8-aminonaphthalene-1,3,6-trisulphonic acid sodium salt (ANTS), and 45 mM *p*-xylenebis(pyridinium)bromide (DPX), submitted to a series of 10 freeze/thaw cycles and further extruded 11 times on a Thermobarrel extruder through polycarbonate filters (Nucleopore) with a diameter of 100 nm. These vesicles were applied onto a Sephadex *G*75 column and eluted with 5 mM Hepes (pH 7.4) containing 100 mM NaCl to remove the unencapsulated ANTS and DPX. The final concentration of phospholipid was determined by phosphorus analysis [43].

### 3.3. ANTS/DPX Leakage Assays

Dequenching of co-encapsulated ANTS and DPX fluorescence in LUVs (obtained as described in Section 2.2) was measured to assess the release of vesicular contents to the medium, as described in the literature [44]. Briefly, a lipid suspension was diluted in 5 mM Hepes (pH 7.4) containing 100 mM NaCl to give a final lipid concentration of 0.1 mM. Different aliquots of Pln149a and Pln149S (0.5 to 16 μM) were added to the cuvette, and the extent of leakage was measured for all peptide-lipid ratios. Fluorescence intensities were recorded at 25 °C with continuous stirring in the cuvette medium to allow the rapid mixing of peptide and vesicles. Measurements were performed in an AMINCO-Bowmann Series 2 Luminescence Spectrometer (Madison, WI, USA) by setting the ANTS emission at 520 nm as a function of time and the excitation at 355 nm. The percentage of leakage was calculated using the following Equation:

(1) $${\%}_{Leakage}=\frac{\left(F-F_{0}\right)}{\left(F_{100}-F_{0}\right)}\times 100$$

where *F*_0_ is the initial fluorescence intensity of vesicles, *F* is the fluorescence intensity achieved after adding the peptide, and *F*_100_ is the measured fluorescence after the addition of Triton X-100.

### 3.4. Far-UV Circular Dichroism (CD)

The CD spectra of Pln149a and Pln149S (both at 0.15 mg/mL) were recorded from 190 to 250 nm as the average of 8 scans in the presence of different phospholipid vesicles on a J-815 spectropolarimeter (Jasco Instruments, Tokyo, Japan) using a 0.1 cm path length rectangular quartz cuvette. The peptides were incubated for 10 min with LUVs of pure dipalmitoyl-phosphatidyl-choline (DPPC), pure DPPG and a mixture of DPPC and DPPG (at 3:1, 1:1 and 1:3 molar ratios) at a 1:20 peptide/lipid ratio (mol/mol).

### 3.5. Tryptophan Fluorescence Titrations

The intrinsic fluorescence emission spectrum from the Trp residue in Pln149W and Pln149SW (1 μM) in 5 mM Hepes (pH 7.4) was recorded from 310 to 450 nm at 25 °C, using a 1 cm path length quartz cuvette under continuous stirring and excitation at 295 nm. Incremental additions of pure DPPG LUVs were titrated into the initial solution at 25:1, 50:1, 100:1, 200:1, 300:1, 400:1, and 500:1 lipid/peptide ratios (mol/mol), and the fluorescence spectrum was recorded at each condition after 5 min of incubation. Fluorescence intensities were corrected for the contribution of light scattering by subtracting the vesicle reference spectra and for the dilution effect with a parallel lipid titration using *N*-acetyl-tryptophan-amide (NATA) [45]. Origin Pro 7.5 software was used to determine the apparent dissociation constants (*k*) by fitting the data from fluorescence intensity at 350 nm into a hyperbolic equation, as follows:

(2) $$F/F_{0}=\frac{1+[L][(F_{\text{max}}/F_{0})-1]}{k+[L]}$$

where *F* is the fluorescence intensity at each lipid concentration, *F*_0_ is the initial fluorescence intensity, *F*_max_ the fluorescence at the end of the titration, and [L] the total lipid concentration [46]. The apparent binding constant (*k*) was used to determine the real binding constant *K*:

(3) K=W/k

where, *W* is the molar concentration of water (55.3 M). Then, the *K* value of each peptide was used in the relation given below to calculate the fraction of peptide bound to the liposomes (*F_x_*):

(4) $$F_{x}=\frac{K\;\;[L]}{W+K[L]}$$

where, [*L*] is the concentration of lipid on each point of the titration.

### 3.6. Isothermal Titration Calorimetry (ITC)

The total binding enthalpy of Pln149a and Pln149S on DPPG liposomes was investigated by the ITC technique using a high sensitivity VP-ITC MicroCalorimeter (MicroCal LLC, Northampton, MA, USA) with a reaction cell volume of 1.417 mL. LUVs of DPPG (10 mM) were prepared in 10 mM Hepes (pH 7.4) and placed in the calorimetry cell, and a solution of each peptide (100 μM) was injected through the titration syringe in 5 aliquots of 10 μL each at 25 °C and 300 rpm. Prior to use, solutions were degassed for 10 min under moderated vacuum (ThermoVac, Northamptom, MA, USA) at 20 °C. In control experiments, the corresponding peptide solution was injected into the Hepes buffer. The data were acquired by software developed by MicroCal, and the heats of dilution were subtracted from the actual measurements, using Origin 7.0 software.

### 3.7. Hemolytic Activity

Aliquots (100 μL) of 1% (*v*/*v*) suspension human red blood cells in 20 mM Sodium Phosphate buffer (pH 7.4) with 0.15 M NaCl were incubated for 1 h at 37 °C with the peptides Pln149a and Pln149S in serial dilutions (500 to 1 μM) to determine the hemolytic effect of the peptides. After incubation, samples were centrifuged at 3000× *g* and the supernatant was evaluated spectrophotometrically at 405 nm. The percentage of hemolysis was calculated using the Equation (5), where Triton X-100 was used as control of 100% lysis:

(5) $${\%}_{hemolisis}=\frac{\left[A_{Peptide}-A_{PBS}\right]}{A_{Triton}-A_{PBS}}$$

### 3.8. Antibacterial Activity and MIC Determination

The antibacterial activities of Pln149a and Pln149S against *Staphylococcus aureus* ATCC 25923 and *Pseudomonas aeruginosa* ATCC 9027 were investigated with broth growth inhibition assays. Growth inhibition assays were carried out with sterile Pln149a and Pln149S (620 to 1.2 μM) in PBS (pH 7.4) incubated 1:1 (*v*/*v*) with a bacterial suspension of each of the bacteria (10^6^ UFC/mL) in Mueller-Hinton broth on a 96-well microtiter plate. The microplates were incubated for 24 h, at 37 °C and growth inhibition was analyzed at 600 nm on a Microplate TP-reader (Thermoplate, Palm City, FL, USA). A positive control with PBS and a negative control with 0.4% (*v*/*v*) formaldehyde were employed. Minimal inhibitory concentration (MIC) was considered the lowest concentration that inhibits 90% bacterial growth. All experiments were performed in duplicate. After 24 h incubation, to determine the Minimum bactericidal concentration (MBC) of Pln149a and Pln149S, aliquots (5 μL) of each well were subcultivated in Petri plaques with Mueller-Hinton agar in the absence of the peptides for 24 h.

## 4. Conclusions

Our results demonstrate that the binding of the antimicrobial peptide Pln149a to liposomes should require a strong electrostatic attraction with the negatively charged surface, in agreement with the hypothesis described for the antimicrobial peptide PlnA, in which the positively charged residues of the peptide and the negatively charged groups in the membrane are important for structuring the peptide and for its functionality. This is an important step of the binding that can determine the specificity of the peptide. The peptides Pln149a and Pln149S, for instance, presented stronger antimicrobial action against *S. aureus*, a Gram-positive bacteria, which presents about 60% content of PG and 40% of cardiolipin and lysophosphatidylglycerol at the cell membrane [47].

Moreover, the binding of the amphipathic peptide Pln149a to the phospholipids was significantly affected with the substitution of the Tyr1, reducing the leakage properties of the peptide, decreasing the amount of peptide bound to the surface of the liposomes, and resulting in a weaker binding of the peptide to the vesicles. It can be concluded that Pln149a plays its antimicrobial activity on cellular membranes driven by interactions established with two different regions of the peptide: the amphipathic helix which is associated with the phospholipid headgroups, and the residues at the *N*-terminus, particularly Tyr1 which showed to contribute in the membrane binding, especially in the lipid-aqueous interface which is a preferential location for the Tyr residue in membrane.

## Supplementary Information



## Figures and Tables

**Table 1:** Fraction of bound peptide at the surface of DPPG liposomes.

Peptide/lipid	Pln149W (%)	Pln149SW (%)
(1:25)	88.8	75.6
(1:50)	94.1	86.1
(1:100)	96.9	92.5
(1:200)	98.4	96.1
(1:300)	99.0	97.4
(1:400)	99.2	98.0
(1:500)	99.4	98.4

**Table 2:** MIC and MBC of Pln149a and Pln149S.

	*S. aureus*		*P. aeruginosa*	

Peptide	MIC *	MBC *	MIC	MBC
Pln149a	19	78	155	155
Pln149S	19	155	155	310

* MIC and MBC values in Mm.
