# Supplementary material for: Contribution of the Tyr-1 in Plantaricin149a to Disrupt Phospholipid Model Membranes
Source: Int J Mol Sci. 2013 Jun 7;14(6):12313–28. doi: 10.3390/ijms140612313 (PMC3709787; doi:10.3390/ijms140612313)
Supplement: Supplementary file 1 [file ijms-14-12313-s001.pdf]

Supplementary Information

Figure S1. Reverse Phase Chromatography of the four analog peptides (**a**) Pln149a; (**b**) Pln149S; (**c**) Pln149W; (**d**) Pln149SW. Column was equilibrated with H_2O (TFA 0.1%) and eluted in a gradient of acetonitrile 90% (TFA 0.1%) in 40 min (dotted curves), flow rate of 1 mL/min.

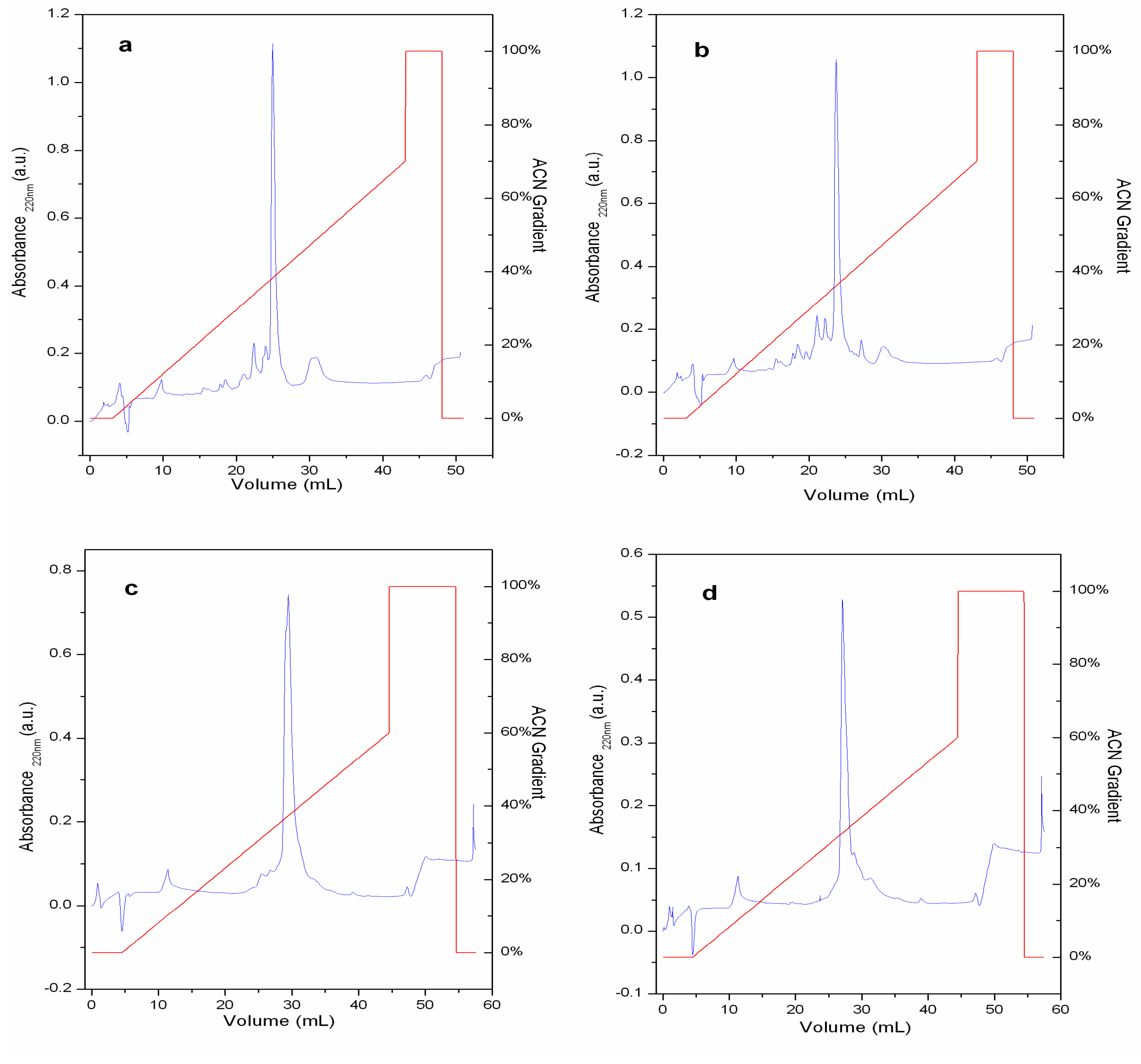

Figure S2. Leakage assays of Pln149a and Pln149S. (**a**) The internal content of DPPG was released by the action of Pln149S (1 to 16 μM). Leakage action of (**b**) Pln149a (0.5 to 2 μM) and (**c**) Pln149S (1 to 3 μM) on POPG liposomes.

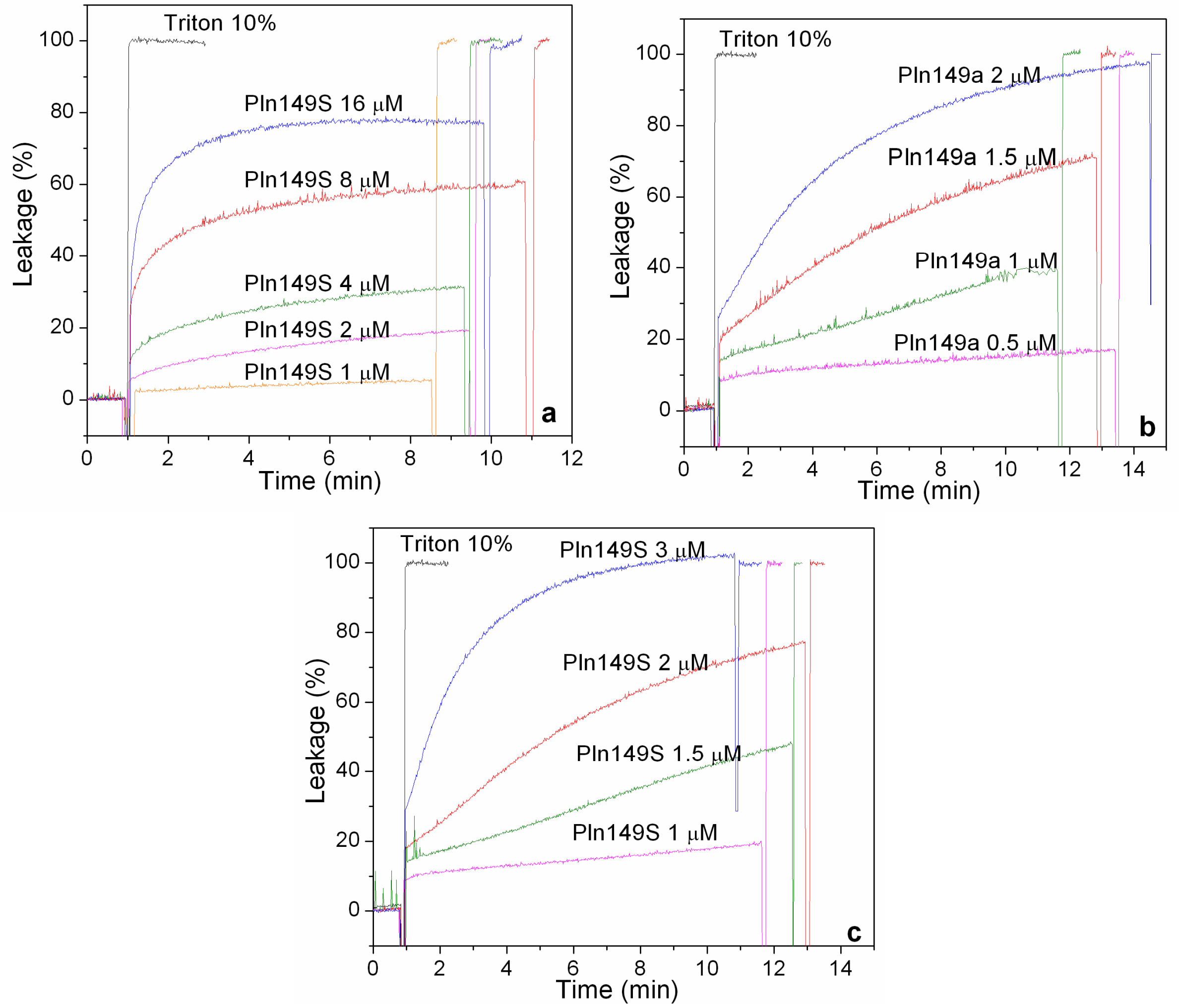

© 2013 by the authors; licensee MDPI, Basel, Switzerland. This article is an open access article distributed under the terms and conditions of the Creative Commons Attribution license (http://creativecommons.org/licenses/by/3.0/).
